# DNA Methylation Profiling of rAAVs Reveals Hypomethylated Genomes and Evidence for Predominant Packaging of Replicated Genomes

**DOI:** 10.3390/v18101158

**Published:** 2026-10-18

**Authors:** Julia Manz, Julia Woernle, Juergen Hubbuch, Rohan Deepak, Florian Noack, Fabian Huellen

**Affiliations:** 1 Gene Therapy Technical Development, Roche Diagnostics GmbH, 82377 Penzberg, Germanyfabian.huellen@roche.com (F.H.); 2 Institute of Process Engineering in Life Sciences, Section IV: Biomolecular Separation Engineering, Karlsruhe Institute of Technology, 76131 Karlsruhe, Germany; 3 Nucleic Acid Based Medicine, Large Molecule Research, Therapeutic Modalities, Roche Pharma and Early Research, Roche Diagnostics GmbH, 82377 Penzberg, Germany

**Keywords:** gene therapy, rAAV, adeno-associated virus, CpG methylation

## Abstract

Immune responses to recombinant adeno-associated viral vectors (rAAVs) impair the therapeutic potential of gene therapies. One trigger for these responses upon systemic administration is the activation of Toll-like receptor 9 by unmethylated cytosine–phosphate–guanine motifs within the vector genome. To better understand the epigenetic profile of rAAVs, we performed bisulfite sequencing on vector genomes produced either via standard triple transfection or via a stable cell line. Furthermore, we investigated whether the epigenetic state of the template DNA influences the encapsidated genome by using in vitro-methylated plasmids during triple transfection. Our results demonstrate that, regardless of the production platform, rAAV genomes lack substantial CpG methylation. Notably, even when a fully CpG-methylated template plasmid was used, the resulting vector DNA exhibited only 4.56% methylation, indicating that alternative strategies—beyond modifying the template DNA—are required to reduce CpG-associated immunogenicity. Additionally, comparing the methylation patterns of rAAVs generated from GpC- and CpG-methylated plasmids revealed that vector genomes were predominantly packaged following genome replication rather than directly from the plasmid DNA. These findings provide critical insights into rAAV packaging mechanisms and inform future strategies for vector engineering.

## 1. Introduction

Adeno-associated viruses (AAVs) consist of a 25-nm icosahedral capsid containing a single-stranded DNA (ssDNA) payload of approximately 4.7 kilobases (kb) [1]. The coding sequence of the genome is flanked by inverted terminal repeats (ITRs) that are crucial for viral replication and packaging [2,3]. Several advantages, such as non-pathogenicity and their ability to infect both dividing and non-dividing cells, make them promising candidates for gene therapy [4,5]. To obtain a recombinant AAV vector (rAAV), all viral sequences except the ITRs are removed and replaced with the desired transgene cassette. To date, rAAV-based therapeutics are extensively explored in clinical trials and several of these drugs have already been approved [4,6].

The most common rAAV production method is the so-called triple transfection (TTF) of HEK293 cells. To this end, three different plasmids are co-transfected: (i) a vector plasmid that encodes the transgene flanked by ITRs (pITR), (ii) a plasmid providing the *rep* and *cap* genes (pRepCap), and (iii) a helper plasmid containing the adenoviral helper genes (pHelper) [7]. To decrease manufacturing costs and increase reproducibility, the use of stable cell lines (SCLs) represents a promising alternative. Several types of SCLs exist, for instance, packaging cell lines containing the *rep/cap* construct integrated into their genomes, producer cells harboring the rAAV genome (transgene flanked by ITRs), or ‘all-in-one’ systems that have all necessary functions integrated [8,9].

Despite rAAVs being the vehicle of choice for in vivo gene therapy, immune responses against the rAAV capsid and the delivered nucleic acids negatively impact their overall therapeutic profile [4,5,10]. Pre-existing antibodies, complement activation, innate immune receptors including Toll-like receptor (TLR) 2 and TLR9, and the presentation of transgene or capsid peptides on major histocompatibility complex I (MHC-I) or MHC-II can all interfere with the therapy [4]. ITRs are known for their high CpG content and studies indicate that codon optimization leads to an increase in CpG motifs [11,12]. In contrast, apart from CpG islands, the mammalian genome is generally CpG-poor and methylated. Unmethylated CpGs are recognized by TLR9 and trigger inflammation and thus an antiviral response via the MyD88 pathway [13,14,15]. To avoid TLR9 activation, CpG depletion is one option that is under investigation [16,17]. For instance, Pan et al. were able to produce functional vectors with CpG-free ITRs; however, the vector yield was decreased by approximately threefold most likely due to the ITRs’ critical role in genome replication [18].

Since TLR9 recognizes unmethylated CpGs, not only the presence of this dinucleotide but also its epigenetic modification is crucial. Nevertheless, very few publications address this topic. To bridge this gap, in the current study, we compared the influence of different rAAV production strategies, namely TTF versus the use of an SCL, on the methylation state of the vectors. Moreover, we investigated whether in vitro methylation of plasmids influences the epigenetic signature of the produced rAAVs.

## 2. Materials and Methods

### 2.1. rAAV Production via Triple Transfection

#### 2.1.1. Plasmid Preparation

To evaluate the influence of the methylation state of the pITR on that of the produced rAAVs, differentially methylated forms of the plasmid were generated. CpG methylation was carried out using M.SssI (NEB, Ipswich, MA, USA) and GpC methylation using M.CviPI (NEB, Ipswich, MA, USA) according to the manufacturer’s protocol. A total of 16 units (U) of methyltransferase were used for 4 µg of plasmid DNA. The reaction mix was incubated at 37 °C for 4 h. Due to its instability, additional S-adenosylmethionine (SAM) was added after 2 h. After heat inactivation of the enzyme, the DNA was purified using Pellet Paint Co-precipitant (Novagen, Merck Millipore, Darmstadt, Germany) according to the manufacturer’s instructions. To verify successful methylation, restriction digestions using MspI, HpaII, and HaeIII (NEB, Ipswich, MA, USA) were performed.

#### 2.1.2. rAAV Production

rAAVs were produced using a standard triple transfection protocol. Briefly, suspension HEK293 cells (provided by Roche Diagnostics GmbH, Penzberg, Germany) were transfected with pHelper, pRepCap, and methylated or unmethylated pITRs. Transfections were performed in triplicate per pITR in 125-mL shake flasks. After purification via affinity chromatography using an AAVX resin, the genomic titer was determined using dPCR, and capsid titers were measured with Electrochemiluminescence Immunoassay (ECLIA).

### 2.2. rAAV Production Using a Stable Cell Line

Proprietary stable HEK293 cells containing an integrated pITR in the adeno-associated virus integration site 1 (AAVS1), obtained from Cornelius Frank (Roche Diagnostics GmbH, Penzberg, Germany), were cultured in suspension in a commercially available expression medium at 37 °C with 5% CO_2_. Cells were transiently transfected with pHelper and a wild-type AAV9 pRepCap using a commercial transfection reagent. Three days after transfection, cells were lysed by adding a non-ionic detergent and an endonuclease to digest residual plasmid and host cell DNA. The lysate was clarified by salt adjustment and centrifugation. Subsequently, rAAV particles were concentrated from the clarified supernatant via polyethylene glycol precipitation. The next day, rAAVs were pelleted by centrifugation and resuspended in DPBS supplemented with 0.001% Poloxamer 188. Following DNase I and Proteinase K (NEB, Ipswich, MA, USA) digestion, the vector genome titer was determined by digital polymerase chain reaction (dPCR) using the QIAcuity Eight Digital PCR System (Qiagen, Hilden, Germany).

### 2.3. DNA Extraction

Genomic DNA (gDNA) from the stable cell line was extracted using the DNeasy Blood and Tissue Kit (Qiagen, Hilden, Germany) according to the manufacturer’s protocol. rAAV DNA samples were treated with DNase I (NEB, Ipswich, MA, USA), and the viral DNA was extracted with the QIAamp MinElute Virus Spin Kit (Qiagen, Hilden, Germany). The manufacturer’s protocol was followed, except that the protease digestion was extended to 25 min.

### 2.4. Bisulfite Sequencing

Plasmid, genomic, and rAAV DNA were bisulfite-converted using the EZ DNA Methylation-Gold Kit (Zymo Research, Irvine, CA, USA) according to the manufacturer’s instructions. Plasmid DNA and rAAV DNA generated by TTF were amplified using primer pairs 1–7 (Table 1). Since the SCL did not contain binding sites for primer pair 7, only amplicons 1–6 were generated.

Q5U Hot Start High-Fidelity DNA Polymerase (NEB, Ipswich, MA, USA) was used for DNA amplification. The protocol consisted of an initial denaturation step at 98 °C for 10 s, 25 cycles at 98 °C for 10 s, 58 °C for 20 s, and 68 °C for 30 s, followed by a final extension step at 68 °C for 5 min. PCR amplicons were purified with 1× volume/volume AMPure XP Beads (Beckman Coulter, Brea, CA, USA) according to the manufacturer’s instructions. Amplicons derived from the same DNA sample were pooled in equimolar ratios and underwent library preparation using the NEBNext Ultra II DNA PCR-free Library Prep Kit for Illumina (NEB, Ipswich, MA, USA) according to the manufacturer’s protocol. The sequencing run was performed on a NextSeq 2000 (Illumina, Inc., San Diego, CA, USA).

### 2.5. Sequencing Data Analysis

Raw base call (BCL) files were demultiplexed and converted to FASTQ format using the BCL Convert software (version 4.2.11, Illumina), allowing one mismatch in the index sequences. Automatic adapter trimming was performed during conversion using the standard Illumina adapter sequences for Read 1 (5′-AGATCGGAAGAGCACACGTCTGAACTCCAGTCA-3′) and Read 2 (5′-AGATCGGAAGAGCGTCGTGTAGGGAAAGAGTGT-3′), with a minimum overlap of one base. Reads shorter than 35 base pairs after adapter trimming were discarded. Using Cutadapt (version 5.2) [19], the first and last 25 base pairs were trimmed to correct for methylation bias. The trimmed reads were aligned to the reference genome using Bismark (version 3.0.0), with Bowtie 2 (version 2.5.5) as the underlying alignment algorithm [20] and the option --score_min L,0,−0.3. Subsequently, methylation levels were extracted using the Bismark_methylation_extractor tool with the option --CX to obtain data for cytosines in the CpG-context and with --comprehensive to combine the data from the forward and reverse reads. The final cytosine report was generated using the tool coverage2cytosine with the options --nome-seq and --coverage_threshold 10. Since the primers used for PCR (Table 1) were designed to bind exclusively to the forward strand, only reads derived from this strand were considered further.

## 3. Results

### 3.1. Triple Transfection-Derived rAAVs Are Virtually Unmethylated

TTF using HEK293 cells is the most common method for rAAV production. To investigate the methylation state of the vectors generated and to examine the influence of the epigenetic state of the pITR, three different conditions were tested. In the first condition, native plasmids (pITR, pRepCap, and pHelper) were used for TTF. In the second, the pITR was CpG-methylated prior to transfection, and in the third, a GpC-methylated pITR was used. Since DNA methyltransferases (DNMTs) methylate CpGs in mammals [21], methylation of rAAVs produced using the CpG-methylated plasmid could be due to either direct packaging from the plasmid or de novo methylation of the vector genome resulting from rolling hairpin replication. To distinguish between these two scenarios, the GpC-methylated pITR was used, as no GpC methylation occurs in humans.

**Figure 1:**
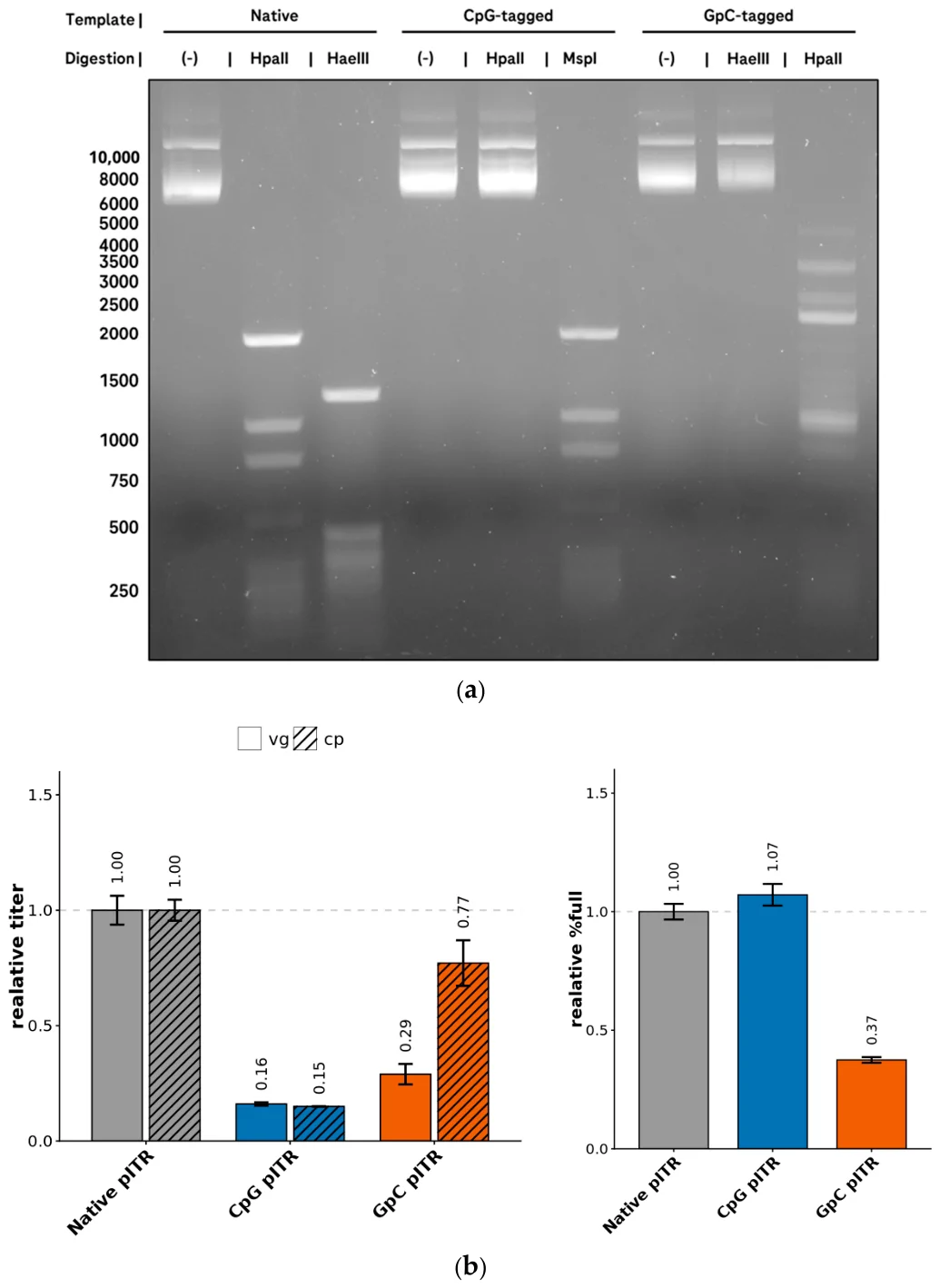
Generation of rAAVs by TTF of differentially methylated pITRs. Native, CpG-, or GpCmethylated pITRs were used for TTF. (**a**) Restriction digestion of the pITRs. HpaII is sensitive to CpG methylation, and HaeIII cannot cleave GpC-methylated DNA. (**b**) Genomic and capsid titers of the produced rAAVs were measured using dPCR and ECLIA, respectively. The production was performed in triplicate, and values were normalized to those of the native pITR. Data are presented as normalized means ± standard deviation.

Successful plasmid methylation was verified via restriction digestion using the CpG methylation-sensitive enzyme HpaII [22] and the GpC methylation-sensitive enzyme HaeIII [23] (Figure 1a). CpG methylation decreased both genomic and capsid titers to approximately 15% (CpG pITR: 2.57 × 10^10^ vg/mL, 1.90 × 10^11^ cp/mL; native pITR: 1.57 × 10^11^ vg/mL, 1.25 × 10^12^ cp/mL). In contrast, GpC methylation reduced the genomic titer to about 29%, but the capsid titer to only approximately 77% (GpC pITR: 4.61 × 10^10^ vg/mL, 9.88 × 10^11^ cp/mL; native pITR: 1.57 × 10^11^ vg/mL, 1.25 × 10^12^ cp/mL). Consequently, the full-to-total ratio remained unchanged for the CpG sample but was reduced to approximately 37% for the GpC sample (Figure 1b).

The methylation states of the plasmids used and the produced rAAVs were determined by bisulfite sequencing. The results are summarized in Table 2, and sequencing quality attributes in Table A1. The manual for the EZ DNA Methylation-Gold Kit used for bisulfite conversion states a conversion efficiency of >99% and >99% protection of methylated cytosines [24]. Therefore, values of less than 1% methylation are likely attributable to technical background noise rather than a true biological signal. As expected, the native pITR did not show any methylation, and the produced rAAVs were also unmethylated. As already indicated by the restriction digestion (Figure 1a), GpC methylation of the pITR was successful (96.34%). The slightly elevated CpG methylation detected (6.90%) is in line with the off-target activity at CCG sites reported for M.CviPI [25]. Since the generated rAAVs were not methylated, the packaged vector DNA is derived from rolling hairpin replication, and no direct packaging from the plasmid DNA appears to occur. For the CpG sample, methylation of the pITR was successful (98.26%; Figure 1a). The CpGs of the resulting rAAVs were only slightly methylated (4.56%). In combination with the data obtained for the rAAVs of the GpC sample, this suggests maintenance methylation activity by cellular methyltransferases.

**Figure 2:**
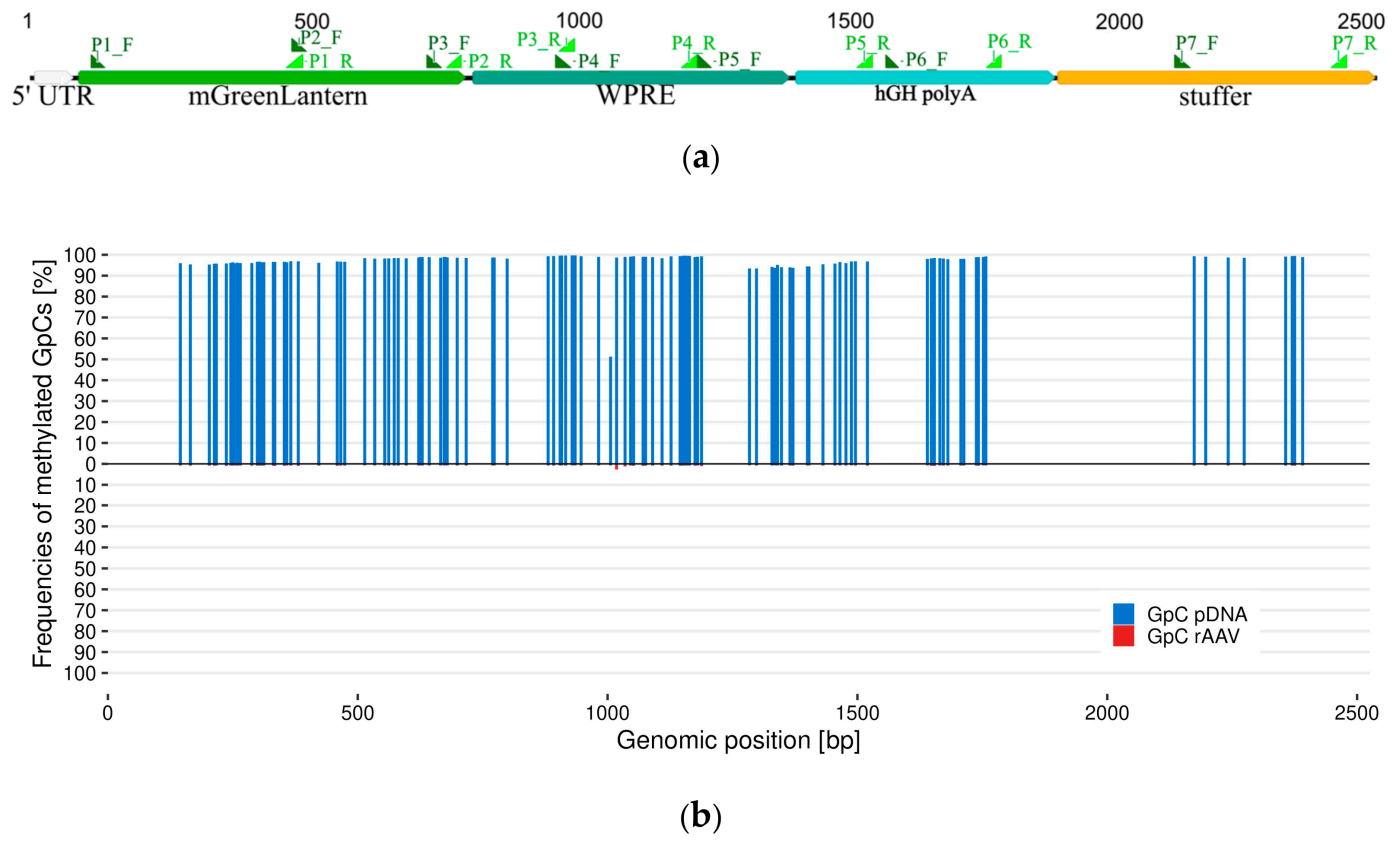

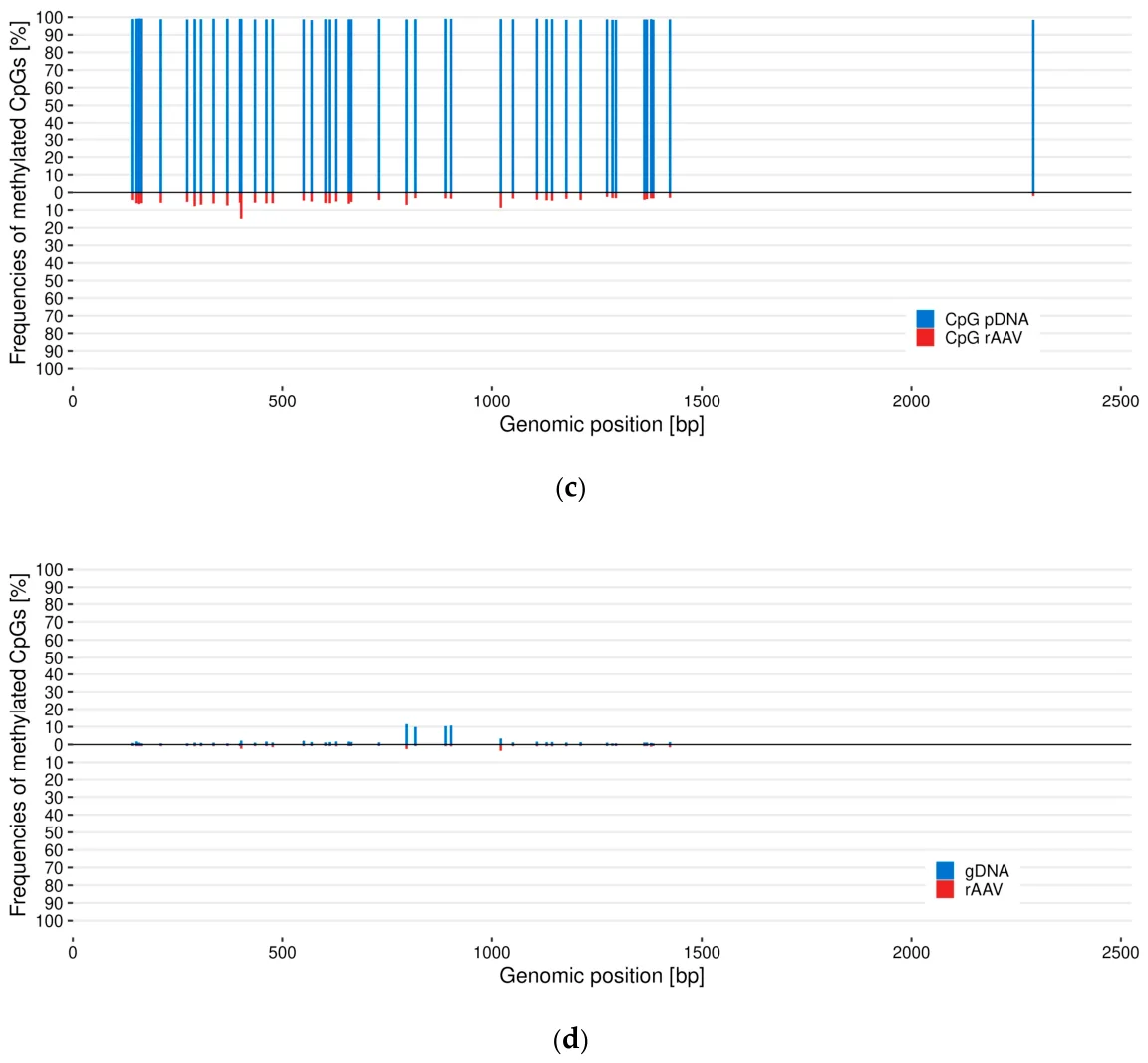
Analysis of the methylation pattern of individual positions. (**a**) Map of the region of the reference vector genome that was amplified by PCR. The binding sites for the different primer pairs are highlighted. For the TTF samples, all seven pairs were used. Due to a slight difference in the stuffer sequence, only pairs 1–6 could be used for the SCL. (**b**–**d**) The x-axis shows the position in the reference. The y-axis shows the percentage of methylated cytosines at this position. Data are shown in a mirrored representation, with those for the pITRs/gDNA plotted upwards (above the baseline) and those for the resulting rAAVs plotted downwards (below the baseline). Only positions with a coverage of at least 1% of the median coverage for the respective sample are shown. (**b**) Results for GpC-methylated pITRs, (**c**) CpG-methylated pITRs, and (**d**) SCL samples.

Next, we examined the methylated positions to identify any specific patterns or anomalies. Neither the GpC-methylated sample (Figure 2b) nor the CpG-methylated one (Figure 2c) exhibited any distinct hotspot regions. For the rAAV genomes generated with the CpG-methylated pITR, only one position in *mGreenLantern* showed >10% CpG methylation (14.37%).

### 3.2. rAAVs Produced in a Stable Cell Line Are Not Methylated

As rAAVs produced via TTF did not show any substantial methylation, regardless of whether the template was methylated or not, we investigated the methylation profile of rAAVs produced in an SCL. To this end, a cell line with an integrated pITR was transfected with pHelper and pRepCap, and the gDNA and rAAV DNA were bisulfite-sequenced. In the gDNA sample, 1.65% of the cytosines were CpG-methylated and 1.18% GpC-methylated (Table 2). The latter result might be explained by the slight methylation in non-CpG contexts observed in human cells [26,27]. Detailed analysis of the CpG-methylated positions revealed four that were approximately 10% CpG-methylated. One was located in *mGreenLantern*, one between *mGreenLantern* and the WPRE, and the remaining two in the WPRE (Figure 2d). The rAAVs produced using the stable cell line did not show any substantial methylation and were comparable to those produced via TTF with native pITRs (Table 2).

Taken together, our results indicate that CpGs in rAAVs are generally unmethylated, regardless of the usage of TTF or SCLs, and that the use of methylated template DNA only slightly impacts the methylation state of the produced vector genomes.

## 4. Discussion

While the CpG methylation state of rAAV genomes is of less concern in localized treatments of immune-privileged organs such as the eye, it can play a crucial role in systemically administered gene therapies, as unmethylated CpGs are reported to act as pathogen-associated molecular patterns (PAMPs) that are recognized by TLR9, leading to a type I interferon response that can ultimately result in transgene silencing or immune-mediated clearance of transduced cells [14,15,28]. In this study, we generated initial data on how the production strategy and methylation state of the template DNA influence the methylation pattern of the produced rAAVs.

To evaluate the influence of the methylation pattern of the template, native, CpG-methylated, or GpC-methylated pITRs were used for TTF. While CpG methylation drastically reduced both the genomic and the capsid titers, the influence of GpC methylation was more prominent on the genomic titer (Figure 1b). In contrast, Tóth et al. reported no loss in genomic titer when using CpG-methylated pITRs [29]. However, it should be noted that in that study, adherent instead of suspension cells were used, and the titer was not determined after cell lysis but from supernatant samples. Since AAV2, which was used in that work and also in our study, is not efficiently released into the medium [30], vector genomes measured in the supernatant do not necessarily correlate with vector production but could also be the result of apoptosis. Therefore, their data are not directly comparable to ours. Regarding the results presented herein, it has been reported that after transfection, plasmids rapidly concatemerize via homologous recombination and non-homologous end joining [31,32]. Moreover, CpG methylation can completely silence an adjacent, unmethylated promoter in cis [33]. Thus, it seems possible that in the production runs with the CpG-methylated pITR, the plasmids concatemerized after transfection, and the methylated cytosines interfered with the production of Rep and Cap proteins, resulting in a strong decrease in both capsid and genomic titers. In contrast, plasmid concatemerization in the GpC sample would not inhibit the expression of Rep and Cap proteins, allowing capsids to assemble almost normally. However, methylation of cytosines influences the physical properties of DNA [34], which might interfere with Rep binding, leading to a decrease in the genomic titer.

To determine the methylation state of the encapsidated genomes, bisulfite sequencing was performed. In line with previous reports, rAAVs originating from TTF using native pITRs were unmethylated [29,35]. Since the genomes of the rAAVs produced with the GpC-methylated pITR did not show any methylation (Table 2), they must have been generated via rolling hairpin replication [36] and were not directly packaged from plasmid DNA. Although it could be argued that the ITR methylation interferes with Rep protein binding and therefore impairs direct packaging, one would expect at least some GpC-methylated genomes if direct packaging normally occurs in significant amounts. Nevertheless, low-frequency direct packaging or preferential packaging of unmethylated genomes cannot be entirely ruled out. In contrast, rAAV genomes resulting from CpG-methylated pITRs showed 4.56% CpG methylation (Table 2). Taking into account the aforementioned results of the GpC sample, this is likely not due to direct packaging but could hypothetically be the result of the activity of cellular DNMTs, for instance, DNMT1, which is responsible for methylation maintenance [37]. Our results could indicate that this enzyme copies the methylation pattern of the template plasmid DNA onto the newly synthesized viral DNA strands. However, it might not be fast enough to keep up with the replication speed, resulting in only 4.56% CpG methylation. Direct experimental evidence is currently lacking. Future studies employing DNMT1 inhibitors during transfection or utilizing DNMT1-knockdown cell lines would be an option to experimentally validate whether this specific enzyme is responsible for the observed methylation patterns. Although our data suggests that the methylation state of the template DNA might have some influence on that of the generated rAAV genomes, yielding approximately 5% methylation from an almost 100% methylated plasmid is a very low rate, demonstrating that template methylation does not substantially alter the CpG state of the encapsidated vector DNA. Moreover, considering the reduction in production titers, CpG methylation of template DNA does not appear to be a viable strategy.

Since the production strategy might also influence the methylation state of the rAAV genomes, we investigated vectors produced in an SCL. Bisulfite sequencing of gDNA from the producer cells showed a very low level of CpG methylation of the integrated vector genome (1.65%, Table 2). At first glance, this result was surprising, since Tóth et al. reported hypermethylation of approximately 76% for chromosomally integrated AAV2 DNA [29]. In their study, they used latently infected Detroit 6 cells [38], which are cancer cells harboring an integrated AAV2 genome in the AAVS1 locus [39]. In contrast, we used HEK293 cells, which had not been cultured for a long period after rAAV genome integration into the AAVS1 locus, but only for approximately seven weeks. As CpG methylation increases over time [40], this might explain the observed differences in methylation to some extent. Another, probably more likely explanation could be that the methylation patterns differ between the two cell lines. This hypothesis is corroborated by the finding that AAVS1 is known to have an open chromatin conformation in HEK293 cells [41,42]. Interestingly, the 1.65% CpG methylation was not evenly distributed across the investigated region but showed four hotspots with approximately 10% methylation (Figure 2d). However, visual inspection of the sequences flanking the methylated CpGs did not reveal any obvious motifs that distinguished them from unmethylated CpGs. Similar to the rAAVs produced via TTF using native pITRs, the vectors produced using the SCL were also not CpG-methylated (Table 2). Therefore, there appears to be no difference in the CpG methylation state between the two production strategies.

Taken together, our data indicate that CpGs in rAAV genomes are unmethylated, regardless of whether TTF or SCLs are used for production. Moreover, even if the template DNA is heavily CpG-methylated, this has only a minor effect on the methylation state of the generated vectors. While these findings provide a clear baseline, a limitation of this study is that our analysis was restricted to a single representative vector construct and to mammalian production platforms. Evaluating additional sequence contexts, serotypes, and alternative producer systems, such as insect cell-based platforms (e.g., baculovirus/Sf9, which have been reported to differ from HEK cells in vector genome methylation [43,44]), will be valuable to confirm the universal applicability of these results.

According to our data, strategies other than template methylation are necessary to modify the CpG status of encapsidated genomes. Building upon the systematic work already published in this field, future strategies must be based on existing evidence regarding effective vector engineering. These could include CpG depletion of the vector genome. However, as mentioned previously, CpG depletion in the ITRs was reported to strongly decrease rAAV yield [18], highlighting the need to carefully balance reduced immunogenicity with production efficiency. Alternatively, the literature describes strategies such as incorporating short DNA sequences that inhibit TLR9 into the vector genome. For instance, by including several copies of the telomeric motif TTAGGG in their constructs, Chan et al. were able to decrease the immune response in mice and pigs [45]. In addition, co-administration of antimalarial drugs that have been shown to inhibit TLR9 [46] might be an option. Treating mice with hydroxychloroquine one hour prior to rAAV administration significantly enhanced transgene expression [47]. Ultimately, these findings help clarify the methylation landscape of rAAVs and underscore the necessity of developing multifaceted strategies to address CpG-associated immunogenicity in clinical gene therapy applications.

## Figures and Tables

**Table 1:** Primers used for bisulfite PCR.

Primer Pair	Sequence	Amplicon Length [bp]
1	5′ GTTTATCGGGGTGGTGTTTATTTTGG 3′	393
	5′ CCCAAAATATTACCGTCCTCCTTAAAATC 3′	
2	5′ GAGGACGGTAATATTTTGGGGTATAAG 3′	315
	5′ CAACTCGTCCATATCATATATAATCCC 3′	
3	5′ GATTATATGGTTTTGAAGGAGAGGGTG 3′	274
	5′ CAACGAACCACAACTCCTCATAAAAAAAC 3′	
4	5′ GGTTGTTGTTTTTTTATGAGGAGTTGTGG 3′	236
	5′ CAACCAAAAAAAAAACGATAATTTCCCC 3′	
5	5′ TCGTTTATGTTGTTATTTGGATTTTG 3′	325
	5′ CCACCCCATAATATTATAAAAAAACACC 3′	
6	5′ AAGTTGGGAAGATAATTTGTAGGG 3′	214
	5′CTAACCAATATAATAAAACCCCGTCTC 3′	
7	5′ GATAAAGAGTAAATGATGATTCTGAATGG 3′	320
	5′ GTTAGCTCTCATAAACATATCCACAGTTC 3′	

**Table 2:** Quantification of CpG and GpC methylation levels in plasmid, genomic, and rAAV DNA.

Sample	CpG Methylation [%]	GpC Methylation [%]
Native pITR	0.65	0.63
rAAV (native pITR)	0.61	0.22
GpC pITR	6.90	96.34
rAAV (GpC pITR)	0.71	0.24
CpG pITR	98.26	0.88
rAAV (CpG pITR)	4.56	0.20
gDNA	1.65	1.18
rAAV (SCL)	0.44	0.33

## Data Availability

The raw data supporting the results presented in this study are available from the corresponding author upon request. The rAAV reference sequence has been deposited in GenBank under the accession number QB137839.

## Abbreviations

The following abbreviations are used in this manuscript:

rAAV	Recombinant adeno-associated virus
gDNA	Genomic DNA
pITR	Vector plasmid encoding the transgene flanked by ITRs
pHelper	Plasmid containing the adenoviral helper genes
pRepCap	Plasmid providing the *rep* and *cap* genes
ITR	Inverted terminal repeat
DNMT	DNA methyltransferase
dPCR	Digital polymerase chain reaction
SCL	Stable cell line
AAVS1	Adeno-associated virus integration site 1
kb	Kilobase
ssDNA	Single-stranded DNA
TTF	Triple transfection
TLR	Toll-like receptor
MHC	Major histocompatibility complex
U	Unit
SAM	S-adenosylmethionine
PAMP	Pathogen-associated molecular pattern
ECLIA	Electrochemiluminescence Immunoassay

## Appendix A

**Table A1:** Sequencing quality attributes. Region 1 is covered by primer pair 1. Since the amplicons of primer pairs 2 and 3 overlap, they were combined to form region 2. Regions 3–6 are covered by primer pairs 4–7.

Sample	Average Read Quality	Depth	
Native pITR	37.9	Region 1	793,598
		Region 2	572,705
		Region 3	388,006
		Region 4	994,087
		Region 5	999,624
		Region 6	999,743
rAAV (native pITR)	37.5	Region 1	126,824
		Region 2	444,415
		Region 3	230,076
		Region 4	142,889
		Region 5	374,756
		Region 6	161,481
GpC pITR	38.2	Region 1	807,094
		Region 2	576,425
		Region 3	238,559
		Region 4	998,112
		Region 5	999,790
		Region 6	999,832
rAAV (GpC pITR)	37.5	Region 1	45,352
		Region 2	91,955
		Region 3	60,465
		Region 4	63,043
		Region 5	158,616
		Region 6	98,374
CpG pITR	38.1	Region 1	807,302
		Region 2	570,450
		Region 3	422,082
		Region 4	999,278
		Region 5	998,937
		Region 6	999,794
rAAV (CpG pITR)	37.5	Region 1	50,063
		Region 2	92,790
		Region 3	81,472
		Region 4	67,460
		Region 5	173,377
		Region 6	77,396
gDNA	38.1	Region 1	170,908
		Region 2	566,988
		Region 3	335,924
		Region 4	313,441
		Region 5	999,069
rAAV (SCL)	37.8	Region 1	731,060
		Region 2	567,812
		Region 3	418,309
		Region 4	869,852
		Region 5	999,587
